# Nature and Preoperative Prediction of Difficult Non-Channeled Video-Laryngoscopic Tracheal Intubation in Otorhinolaryngological Surgery: A Retrospective Cohort Study of 1932 Adults

**DOI:** 10.3390/jcm15176814

**Published:** 2026-09-02

**Authors:** Darhae Eum, Hyoung Woo Chang, Myoung Hwa Kim, Jinmok Kim, Wyun Kon Park, Hyun Joo Kim

**Affiliations:** 1 Department of Anesthesiology and Pain Medicine, Anesthesia and Pain Research Institute, Yonsei University College of Medicine, Severance Hospital, Seoul 03722, Republic of Korea; deum@yuhs.ac (D.E.); kjm9689@yuhs.ac (J.K.); wkp7ark@yuhs.ac (W.K.P.); 2 Department of Thoracic and Cardiovascular Surgery, Seoul National University Bundang Hospital, Seongnam-si 13620, Republic of Korea; chang.hyoungwoo@gmail.com; 3 Department of Anesthesiology and Pain Medicine, Anesthesia and Pain Research Institute, Yonsei University College of Medicine, Gangnam Severance Hospital, Seoul 06273, Republic of Korea; kmh2050@yuhs.ac

**Keywords:** airway management, intubation, intratracheal, video laryngoscopy, risk factors, otorhinolaryngologic surgical procedures, intubation, difficult

## Abstract

**Background/Objectives:** Video laryngoscopy improves the glottic view, yet difficult intubation still occurs. Whether it reflects a poor view or arises despite an adequate one is rarely quantified in otorhinolaryngological surgery. We described this pattern and its preoperative predictors. **Methods:** In a single-center retrospective cohort of 1932 adults undergoing otorhinolaryngological surgery with non-channeled video laryngoscopy (2018–2021), difficult intubation was defined a priori as two or more laryngoscopic attempts (blade insertions), a lower bound on difficulty. The glottic view (Cormack–Lehane grade) and intubation time described the difficulty, and predictors were identified by logistic regression. An unweighted count of four anatomical bedside factors, selected on rationale (not data-driven significance) at pre-existing clinical thresholds, was the primary risk score; a five-factor version added a resident operator. **Results:** Difficult intubation occurred in 120 patients (6.2%; 95% CI 5.2–7.4). Most occurred despite an adequate view (64% at Cormack–Lehane grade I–II; 45% at grade I alone); the rate was 4.3% (95% CI 3.5–5.4) versus 29.3% (95% CI 22.5–37.1) for good versus poor views (odds ratio 9.2, 95% CI 6.0–14.0), and difficult intubations took about twice as long (median 90 versus 40 s). Independent predictors were shorter inter-incisor and thyromental distances and a resident operator. The four-factor count graded risk from 4.6% to 12.9% (odds ratio 1.63 per factor; apparent area under the curve 0.596, 95% CI 0.546–0.645, not corrected for optimism; positive predictive value 8.8% at one or more factors), and a five-factor count performed similarly (0.631). Within the good-view subgroup, difficulty appeared to relate more to operator inexperience than to anatomy (adjusted odds ratio 2.48 versus 1.13). **Conclusions:** Difficulty was far more likely with a poor view, but because a good view occurred in 92.4% of patients, most difficult intubations arose despite an adequate one. We hypothesize that difficulty then lies in tube delivery rather than visualization; because the analysis conditions on the view, this needs prospective testing. Discrimination was modest and the count exploratory; these single-center findings require external validation.

## 1. Introduction

Difficult tracheal intubation remains a major contributor to anesthesia-related morbidity, because repeated laryngoscopic attempts increase the risk of airway trauma, hypoxemia, and hemodynamic instability [1,2]. Video laryngoscopy improves glottic visualization and first-attempt success relative to direct laryngoscopy [3,4]. Even so, difficult intubation continues to occur when a video laryngoscope is used, and the preoperative identification of patients at risk therefore remains clinically relevant.

The tools available for this purpose were largely shaped by direct laryngoscopy. Composite scores such as the El-Ganzouri Risk Index, developed in elective surgery [5], and the MACOCHA score, developed for intubation in critically ill patients [6], together with reviews of individual bedside airway tests [7], were established in that era. Studies of preoperative prediction have since accumulated, but they address the video-laryngoscopic question only in part: most video laryngoscopy studies have used hyperangulated devices [8,9,10] or have defined difficulty by the glottic view rather than by the intubation itself [9,11], whereas others have used ultrasound in a direct-laryngoscopy setting [12,13]. Where non-hyperangulated, Macintosh-style video laryngoscopy has been studied, it has generally been in selected populations, such as cervical spine surgery [14], or has used a subjective, operator-reported difficult-airway rating rather than the number of attempts as the outcome [15].

One observation recurs across these settings but has rarely been quantified: with a video laryngoscope the glottis is often clearly seen, yet the tracheal tube can still be difficult to deliver, so that the locus of difficulty appears to shift from visualization to tube delivery, an observation reported in both simulated [16] and critical-care [17] settings. Because hyperangulated and Macintosh-geometry blades differ precisely in this balance between obtaining a view and delivering the tube, the trade-off is central to the current clinical debate over which blade to prefer. The glottic view itself captures video-laryngoscopic difficulty only weakly: the Cormack–Lehane classification discriminates poorly between easy and difficult video-laryngoscopic intubation (area under the curve ≈ 0.68 in a pediatric cohort) [18] and is less accurate than the percentage of glottic opening or the Fremantle score [19], while dedicated tools such as the VIDIAC score rely on intraoperative rather than preoperative findings [20].

What has not been characterized is the preoperative prediction of this phenomenon, using simple bedside variables, in an adult surgical cohort with an attempts-based outcome; because we did not undertake a systematic review, we frame this as a gap defined by the apparent absence of comparable work rather than by an exhaustive search. Otorhinolaryngological surgery offers an informative setting in which to do so: anatomical distortion is common in this population—from head and neck pathology, previous radiotherapy, and surgical scarring—and otolaryngologic surgery is independently associated with difficult video laryngoscopy relative to general surgery (odds ratio 1.89) [8]. The higher event rate makes risk stratification both more consequential and statistically more tractable.

We therefore studied a cohort of adults undergoing otorhinolaryngological surgery with video-laryngoscopic intubation, with two objectives. First, using the glottic view (graded by the Cormack–Lehane classification, a scale devised for direct laryngoscopy [18,19]) and the intubation time that were recorded contemporaneously at the time of care for every patient, we characterized the nature of difficult intubation—whether it reflected difficulty in obtaining a view or difficulty in delivering the tube. Second, we identified simple preoperative bedside predictors of difficult intubation and combined them into a pragmatic cumulative count for risk stratification.

## 2. Materials and Methods

### 2.1. Study Design and Ethics

This was a single-center, retrospective cohort study conducted at Severance Hospital, Yonsei University Health System, Seoul, Republic of Korea. The study was approved by the Institutional Review Board (IRB) of Severance Hospital (approval no. 4-2023-1574; approved 19 January 2024) and was conducted in accordance with the Declaration of Helsinki. Because it involved only a retrospective analysis of de-identified records, the IRB granted a waiver of informed consent. The study is reported in accordance with the STROBE (Strengthening the Reporting of Observational Studies in Epidemiology) statement [21] and, for the prediction-tool component, the TRIPOD (Transparent Reporting of a multivariable prediction model for Individual Prognosis Or Diagnosis) statement, including its update for prediction models using regression or machine learning (TRIPOD + AI) [22,23].

### 2.2. Study Population

We screened consecutive adult patients (≥19 years) who underwent otorhinolaryngological surgery with video-laryngoscopic tracheal intubation under general anesthesia between November 2018 and February 2021. The bedside airway-assessment variables central to this study were documented in a structured preoperative airway-assessment template that was later retired under institutional record-management policy; the cohort therefore spans the period during which these variables were captured in a standardized, retrievable format. Study variables were extracted from this structured template and the electronic anesthesia record by two investigators; because every variable had been recorded contemporaneously at the time of care, its values were fixed before the study and could not be altered by extraction.

Patients were excluded if awake intubation was used as the primary technique, if the first intubation attempt used a device other than a video laryngoscope (e.g., a direct laryngoscope or lightwand), if a tracheostomy was already in place at induction, if airway bougination (bougie dilation of the airway) was performed before intubation, or if any candidate predictor or the number of intubation attempts was not recorded.

### 2.3. Video Laryngoscopes and Intubation Technique

Three video laryngoscopes were in routine clinical use during the study period: the AceScope (Ace Medical, Goyang, Republic of Korea), the UEScope (UE Medical, Hangzhou, China), and the GlideScope (Verathon, Bothell, WA, USA). The choice of device for a given case was left to the attending anesthesiologist and was not randomized. All three are non-channeled devices, in which the tracheal tube is advanced alongside the blade, typically over a stylet, rather than through an integrated tube channel; this non-channeled design is the feature common to the whole cohort. The devices differed in blade curvature, spanning a standard Macintosh profile (AceScope), a blade with a shallow Macintosh base and an approximately 45° angled tip (UEScope [24]), and a steeply angulated blade (GlideScope). Because blade-geometry terminology is not standardized and these designs form a continuum rather than discrete classes, we did not assign a single geometry label to the cohort and instead report outcomes by individual device. Stylet and bougie use—principal determinants of tube delivery—were not recorded in a retrievable form and could not be analyzed.

### 2.4. Outcome and Characterization of the Nature of Difficulty

The primary outcome was difficult intubation, defined a priori as tracheal intubation requiring two or more attempts, an attempt being a single insertion of the laryngoscope blade. This threshold was adopted because repeated attempts are a recognized marker of difficult-airway management and are associated with airway complications [25]. Because an attempt was defined as a blade insertion, repeated passes of the tracheal tube while the blade remained in place counted as a single attempt; the incidence reported here is therefore a lower bound on the frequency of difficult tube delivery. The records did not separately capture the number of tube insertions, so a definition counting both blade and tube insertions, as used in the BOUGIE trial [26], could not be reconstructed from this dataset.

To describe whether difficulty was associated with glottic visualization or arose after it, we used two variables documented for every patient. The video-laryngoscopic glottic view was graded by the Cormack–Lehane classification at the first laryngoscopy by the performing operator, who was not blinded to the number of attempts, with grades I–II defined as a good view and grades III–IV as a poor view; the grade was therefore not independent of the outcome. The time to intubation was recorded in seconds, measured from insertion of the laryngoscope blade to confirmation of tracheal intubation by capnography. A difficult intubation occurring despite a good glottic view was regarded as consistent with difficulty arising after visualization—at the stage of tube delivery. Because the glottic view is a post-induction variable that lies between the preoperative predictors and the outcome, and because no variable recorded the reason for a repeated attempt, this locus was inferred from the combination of an adequate view and repeated attempts rather than directly measured, and is treated throughout as a hypothesis.

### 2.5. Candidate Preoperative Predictors

Candidate predictors, all available before induction, comprised demographic variables (age, sex, body mass index), comorbidities (cervical spine disease, obstructive sleep apnea, head and neck cancer, previous neck scar or radiotherapy), bedside airway examination (modified Mallampati class, inter-incisor distance, thyromental distance, cervical spine mobility, dentition, mandibular protrusion, snoring), and the operator level (resident versus consultant or fellow).

For the two continuous bedside measurements associated with the outcome (inter-incisor and thyromental distance), the binary risk factors for the cumulative count were defined at pre-existing clinical thresholds (Section 2.6); data-derived thresholds obtained with the Youden index were examined only as a sensitivity analysis.

### 2.6. Cumulative Risk-Factor Count

As a secondary, pragmatic tool for bedside risk stratification, we constructed an unweighted cumulative count of binary preoperative factors. Four anatomical items, all available at the bedside before induction, formed the primary count: (i) an inter-incisor distance ≤3.0 cm; (ii) a thyromental distance ≤6.0 cm; (iii) modified Mallampati class III–IV; and (iv) limited mandibular protrusion. For the two continuous measurements we used pre-existing clinical thresholds (3.0 and 6.0 cm) rather than cut-offs derived from these data, so that the count did not depend on values optimized within the cohort. The count was the simple sum of the four items (range 0–4). Because operator level, although independently associated with the outcome, is a feature of the process of care rather than a patient characteristic, it was not part of the primary count; a secondary, process-inclusive count added resident operator as a fifth item (range 0–5).

### 2.7. Statistical Analysis

Normally distributed continuous variables are presented as mean (standard deviation, SD) and skewed variables (intubation time) as median (interquartile range, IQR); categorical variables are presented as number (percentage). Baseline characteristics were compared between the easy- and difficult-intubation groups using Welch’s *t*-test for normally distributed continuous variables, the Mann–Whitney U test for intubation time, and the χ^2^ or Fisher’s exact test for categorical variables; these baseline *p* values are unadjusted and descriptive. Univariable logistic regression estimated the unadjusted association of each candidate predictor with difficult intubation. All candidate predictors with at least five events in the exposed group were then entered together in a multivariable model (13 predictors; approximately nine events per variable); predictors carried by fewer than five exposed events were assessed univariably only.

For the nature-of-difficulty analysis, the difficult-intubation rate was calculated by Cormack–Lehane grade (with Wilson 95% confidence intervals), the proportion of difficult intubations occurring despite a good glottic view was quantified, the good-view versus poor-view contrast was expressed as an odds ratio, and the intubation time was compared between easy and difficult intubations. Within the good-view subgroup, the difficult-intubation rate was compared between resident and consultant or fellow operators using the χ^2^ test, and the anatomical count and operator level were entered together in a logistic model to compare their independent contributions, to test whether, once the view was adequate, difficulty related more to anatomy or to operator inexperience.

For the cumulative count, discrimination was summarized by the apparent (optimism-uncorrected) area under the receiver operating characteristic curve (AUC) with a bootstrap 95% confidence interval (CI), the dose–response relationship by the trend odds ratio per additional factor, and the positive and negative predictive values, sensitivity, and specificity at count thresholds; a decision-curve analysis assessed net benefit across probability thresholds [27]. Internal validation of the underlying logistic model used bootstrap optimism correction (1000 resamples) [28]; because the count’s thresholds were pre-existing rather than fitted, the count’s apparent AUC is reported without optimism correction and interpreted as an upper bound on performance in new patients. Whether the count was linear on the logit scale was examined by comparing the linear-trend model with a model treating the count as a categorical factor (likelihood-ratio test), and the linearity of the continuous predictors was checked by adding quadratic terms; collinearity was assessed by the Pearson correlation between inter-incisor and thyromental distance and by variance inflation factors. Calibration was summarized by the Brier score. A subgroup analysis by head and neck cancer status tested the interaction between cancer status and the count.

Analyses were complete-case: of the 2192 patients screened, 62 (2.8%) were excluded because at least one candidate predictor or the outcome was not documented, and the 1932 analyzed patients had complete data for every candidate predictor and the outcome; the partial records of excluded patients were not retained, so the missing-variable pattern could not be characterized. All tests were two-sided, with *p* < 0.05 considered significant. Analyses were performed in Python 3.9 with the pandas v2.3.3, NumPy v2.0.2, SciPy v1.13.1, and statsmodels v0.14.6 libraries. The statistical analysis code was prepared with the assistance of a generative AI tool (Claude Opus 4.8, Anthropic, San Francisco, CA, USA); all code, outputs, and results were reviewed, validated, and verified by the authors.

## 3. Results

### 3.1. Cohort Characteristics

**Figure 1:**
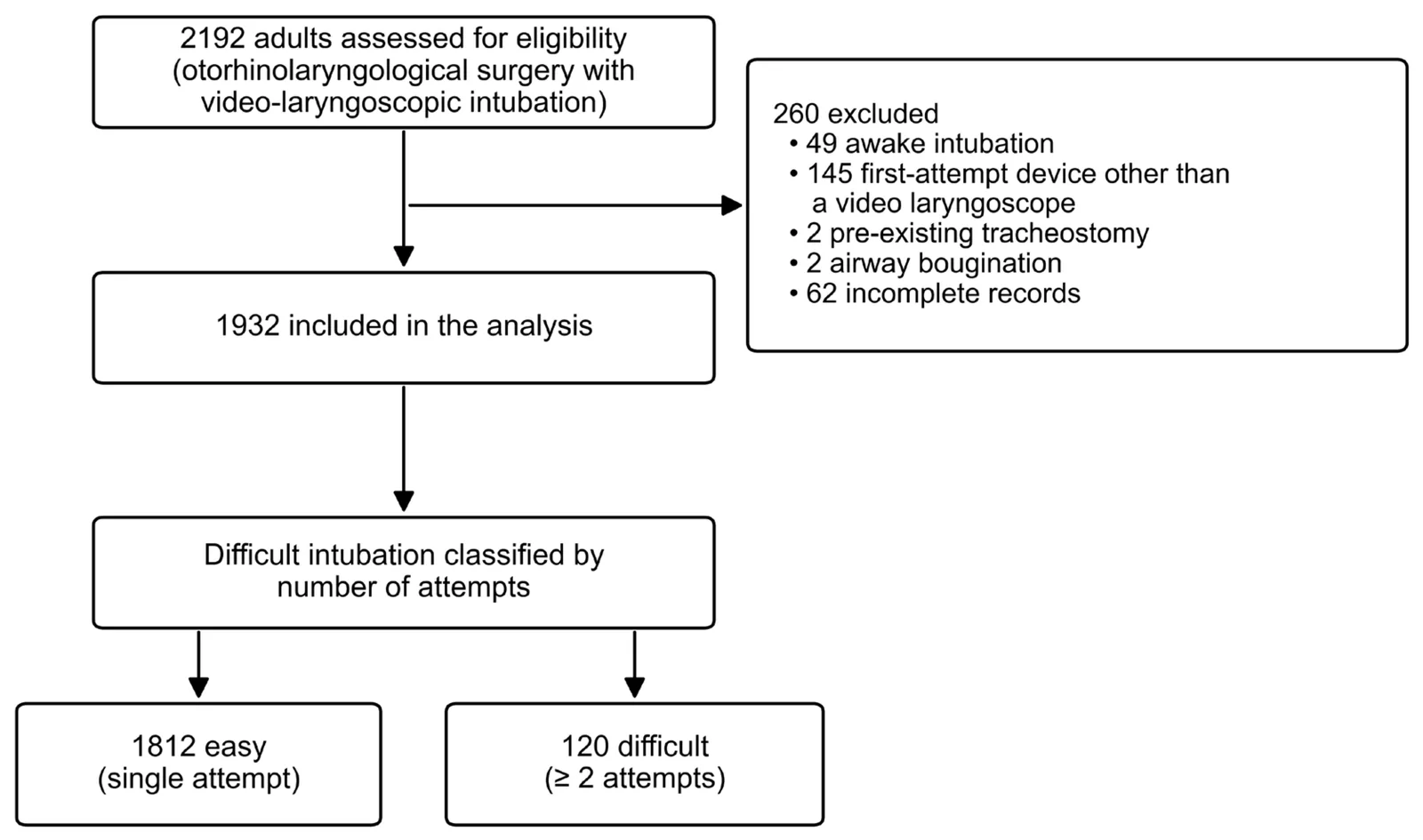
Participant flow. Of the 2192 adults assessed for eligibility, 260 were excluded for the reasons shown, leaving 1932 for analysis; difficult intubation, defined as two or more attempts, occurred in 120.

Of 2192 patients screened, 260 were excluded—49 for awake intubation, 145 for a first-attempt device other than a video laryngoscope, two for a pre-existing tracheostomy, two for airway bougination, and 62 for incomplete records—leaving 1932 (88.1%) for analysis (Figure 1). Surgery involved the nose in 44.5% of patients, the ear in 28.7%, the oral cavity or oropharynx in 21.5%, and the neck in 8.7%; some patients underwent combined procedures, so these categories are not mutually exclusive. All intubations used non-channeled video laryngoscopes (the AceScope, UEScope, or GlideScope). Difficult intubation occurred in 120 patients (6.2%; 95% CI 5.2–7.4): 103 (5.3%) required two attempts and 17 (0.9%) required three. Patient characteristics by intubation difficulty are shown in Table 1.

### 3.2. Nature of the Difficulty: Visualization Versus Tube Delivery

**Figure 2:**
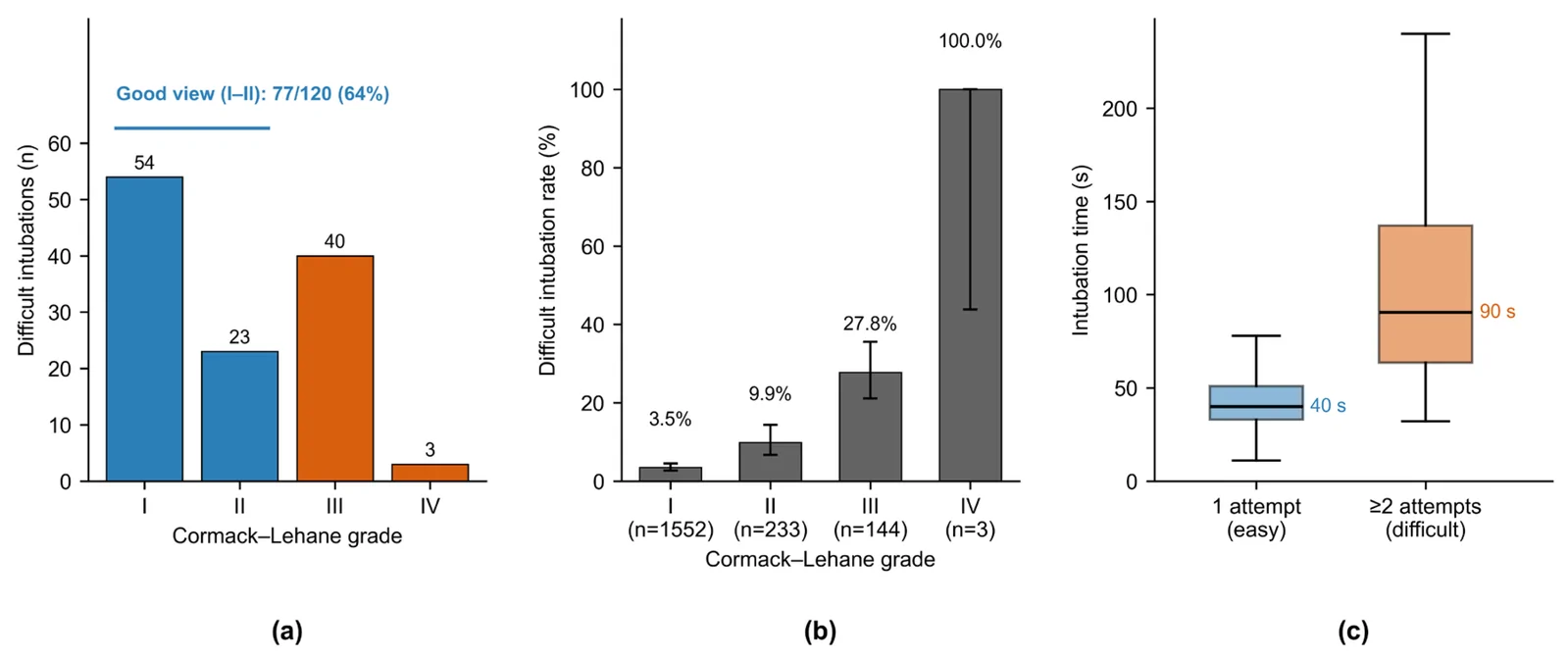
The nature of difficult video-laryngoscopic intubation. (**a**) The distribution of the Cormack–Lehane grade among the 120 difficult intubations; 77 (64%) occurred despite a good glottic view (grade I–II, blue), and 43 (36%) with a poor view (grade III–IV, orange). (**b**) Difficult-intubation rate by Cormack–Lehane grade, with Wilson 95% confidence intervals; the number of patients in each grade is shown below the axis. (**c**) Intubation time in easy (1 attempt) versus difficult (≥2 attempts) intubations; the box plots show the median and interquartile range, with the medians annotated.

A good glottic view (Cormack–Lehane grade I–II) was obtained in 1785 patients (92.4%) and a poor view (grade III–IV) in 147 (7.6%). Of the 120 difficult intubations, 77 (64.2%) occurred with a good glottic view and 43 (35.8%) with a poor view; 54 (45.0%) occurred with a grade I view alone (Figure 2a). The difficult-intubation rate rose as the view worsened—3.5% (54/1552) at grade I, 9.9% (23/233) at grade II, 27.8% (40/144) at grade III, and 3 of 3 at grade IV—although the higher grades comprised few patients (Figure 2b); dichotomized, the rate was 4.3% (77/1785; 95% CI 3.5–5.4) with a good view versus 29.3% (43/147; 95% CI 22.5–37.1) with a poor view (odds ratio 9.2, 95% CI 6.0–14.0). Intubation time was longer in difficult than in easy intubations (median 90 versus 40 s; *p* < 0.001; Figure 2c). Within the good-view subgroup, difficult intubation occurred more often with a resident operator than with a consultant or fellow (36/474 [7.6%] versus 41/1311 [3.1%]; *p* < 0.001).

### 3.3. Preoperative Predictors of Difficult Intubation

In univariable analysis, six preoperative variables were significantly associated with difficult intubation: shorter inter-incisor and thyromental distances, modified Mallampati class III–IV, limited mandibular protrusion, neck scar or radiotherapy, and resident operator (Table 2). The thirteen candidate predictors meeting the entry criterion (Section 2.7) were entered together in the multivariable model, of which three remained independent predictors: inter-incisor distance (adjusted odds ratio [aOR] per cm 0.61, 95% CI 0.45–0.84; *p* = 0.002), thyromental distance (aOR per cm 0.82, 95% CI 0.69–0.97; *p* = 0.019), and resident operator (aOR 1.82, 95% CI 1.21–2.73; *p* = 0.004). Cervical spine disease, neck scar or radiotherapy, and edentulous or denture-wearing each accompanied fewer than five difficult intubations and were assessed univariably only; neck scar or radiotherapy, although univariably associated (OR 6.13, 95% CI 1.18–31.91), rested on only seven exposed patients and was not carried forward to the cumulative count.

### 3.4. Cumulative Risk-Factor Count and Risk Gradient

**Figure 3:**
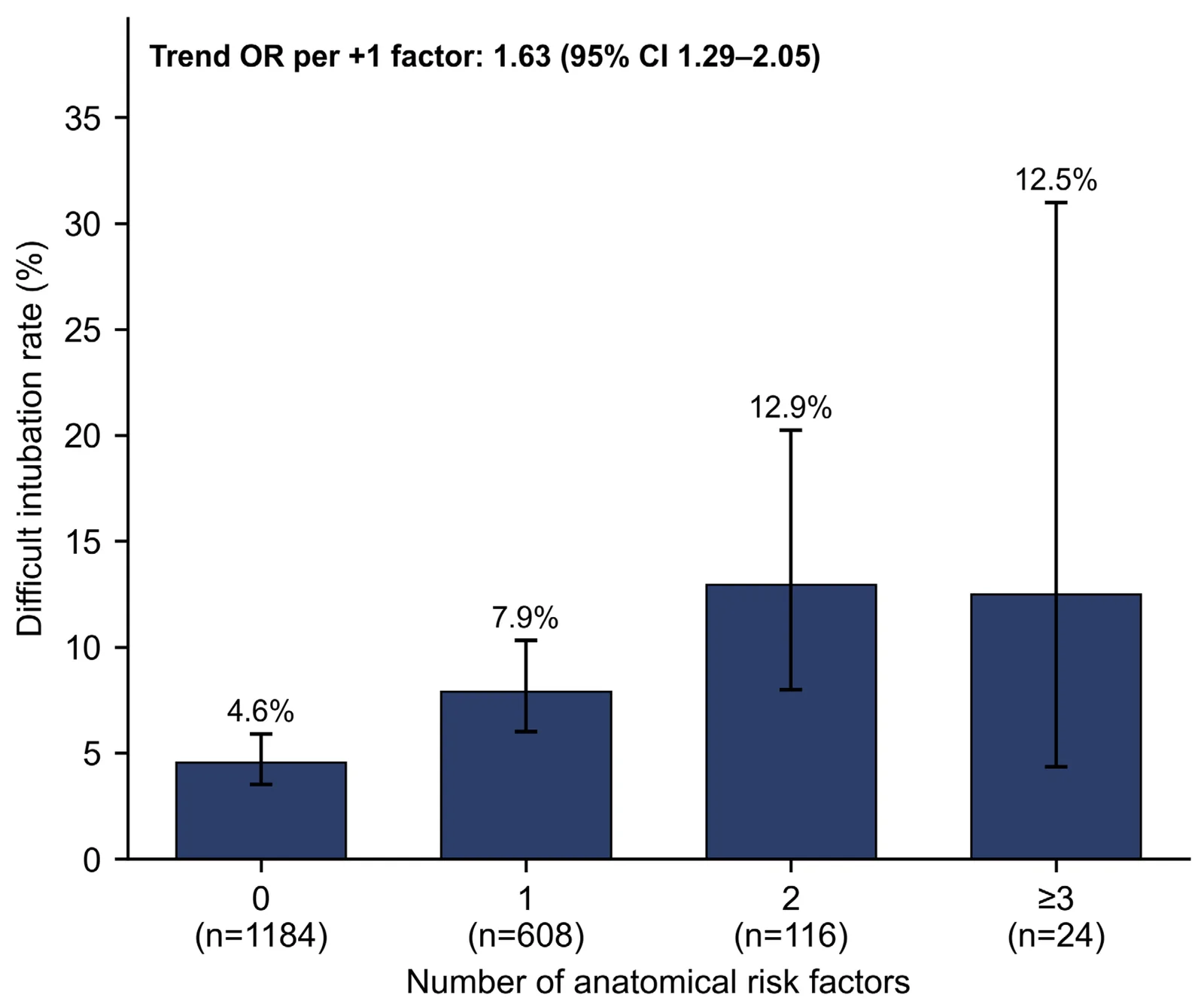
Difficult-intubation rate by the four-factor anatomical count. The bars show the rate with Wilson 95% confidence intervals for each stratum (0, 1, 2, ≥3 factors); the number of patients per stratum is shown below the axis. The trend odds ratio per additional factor was 1.63 (95% confidence interval 1.29–2.05; apparent area under the curve 0.596). The four binary anatomical factors were an inter-incisor distance ≤3.0 cm, a thyromental distance ≤6.0 cm, modified Mallampati class III–IV, and limited mandibular protrusion.

Using the pre-existing thresholds of an inter-incisor distance ≤3.0 cm and a thyromental distance ≤6.0 cm, the four-factor anatomical count showed a graded (dose–response) relationship with difficult intubation, rising from 4.6% (54/1184; 95% CI 3.5–5.9) with no factor to 7.9% (48/608; 95% CI 6.0–10.3) with one factor and 12.9% (18/140; 95% CI 8.3–19.4) with two or more (Figure 3; Supplementary Table S1); the highest strata were sparse (21 patients with three factors and three with four). In Figure 3 the highest group is shown as separate two-factor (12.9%) and three-or-more (12.5%) strata, which are combined as ≥2 in the text because the top strata are sparse. Each additional factor was associated with a 1.63-fold increase in the odds of difficult intubation (95% CI 1.29–2.05; *p* < 0.001), with an apparent (optimism-uncorrected) area under the curve of 0.596 (95% CI 0.546–0.645). At a threshold of one or more factors, the positive predictive value was 8.8% and the negative predictive value 95.4% (sensitivity 55.0%, specificity 62.4%); at two or more factors, the positive predictive value was 12.9% and the negative predictive value 94.3% (sensitivity 15.0%, specificity 93.3%). The high negative predictive value reflects the low prevalence of difficult intubation rather than strong discrimination. Adding resident operator as a fifth, process-related factor gave a slightly higher apparent area under the curve (0.631, 95% CI 0.581–0.678). A count restricted to the three independently associated predictors (inter-incisor distance, thyromental distance, and resident operator) gave a similar apparent area under the curve (0.607).

### 3.5. Internal Validation and Calibration

Bootstrap internal validation of the underlying four-factor logistic model (coefficients in Supplementary Table S2) showed small optimism (apparent area under the curve 0.600, optimism-corrected 0.588). For the deployed unweighted four-factor count, the apparent (optimism-uncorrected) area under the curve was 0.596 (95% CI 0.546–0.645) and should be read as an upper bound on performance in new patients. The Brier score was 0.058, equal to the no-information value at the 6.2% event rate (scaled Brier 0.009); calibration of the count against observed rates is shown in Supplementary Figure S1. In a decision-curve analysis, the count provided a small net benefit only at very low probability thresholds (≤0.075) and none above, so it does not act as a stand-alone decision rule; its role is to flag higher-risk patients before induction rather than to rule difficulty in or out.

### 3.6. Subgroup and Sensitivity Analyses

**Figure 4:**
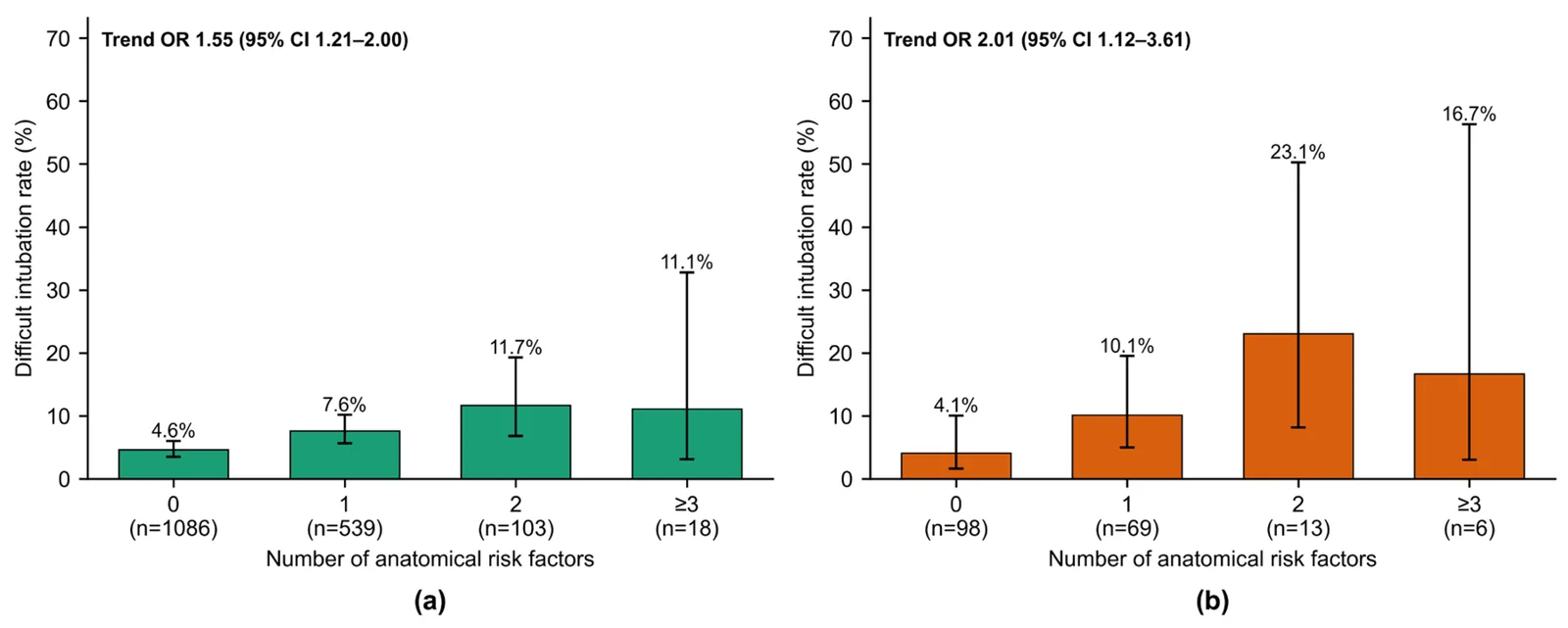
The cumulative risk gradient by head and neck cancer status. The difficult-intubation rate (with Wilson 95% confidence intervals) by the number of anatomical risk factors in patients (**a**) without head and neck cancer (*n* = 1746) and (**b**) with head and neck cancer (*n* = 186). The trend odds ratio per additional factor was 1.55 (95% confidence interval 1.21–2.00) without cancer and 2.01 (95% confidence interval 1.12–3.61) with cancer; the interaction between cancer status and the count was not statistically significant (*p* = 0.42).

The graded association was present across head and neck cancer status: the trend odds ratio per additional factor was 2.01 (95% CI 1.12–3.61) in patients with head and neck cancer (*n* = 186) and 1.55 (95% CI 1.21–2.00) in those without (*n* = 1746), with no significant interaction (*p* = 0.42; Figure 4); the cancer subgroup, with only 15 events across four strata, was too small to establish or exclude a difference. The four-factor count was adequately linear on the logit scale (likelihood-ratio *p* = 0.50 for departure from linearity), so the trend odds ratio summarizes an approximately constant per-factor effect, although the highest strata were sparse; the two continuous predictors were similarly linear (quadratic-term *p* = 0.97 for inter-incisor and 0.25 for thyromental distance), and there was no material collinearity (inter-incisor–thyromental Pearson r = 0.29; all variance inflation factors < 1.2). When the analysis was confined to the good-view subgroup, the difficult-intubation rate rose only weakly with the anatomical count (3.8% [43/1132; 95% CI 2.8–5.1], 5.0% [27/542; 95% CI 3.4–7.2], and 6.3% [7/111; 95% CI 3.1–12.4] for counts of 0, 1, and ≥2); with the anatomical count and operator level modeled together, operator level was a strong independent predictor (adjusted odds ratio 2.48, 95% CI 1.56–3.96; *p* < 0.001) whereas the anatomical count was not (adjusted odds ratio 1.13, 95% CI 0.81–1.57; *p* = 0.48), indicating that difficulty despite a good view related more to operator inexperience than to the anatomical factors. The risk gradient was preserved across alternative inter-incisor and thyromental cut-offs (Supplementary Table S3). The count, subgroup, and sensitivity analyses were exploratory and hypothesis-generating.

A within-cohort comparison of the cumulative count with the modified MACOCHA score and the modified El-Ganzouri Risk Index is reported in the Supplementary Materials (Table S4, Figure S2); the four-factor count (apparent area under the curve 0.596) did not discriminate better than these established scores (El-Ganzouri 0.610, MACOCHA 0.558), all three being modest.

## 4. Discussion

In this cohort of 1932 adults who underwent otorhinolaryngological surgery with non-channeled video laryngoscopy, difficult intubation was uncommon (6.2%). Its character was distinctive: most difficult intubations occurred even though the larynx was well seen—64% at a Cormack–Lehane grade of I–II and 45% at grade I alone—and these intubations took about twice as long as easy ones (median 90 versus 40 s). A simple, unweighted count of four anatomical preoperative factors graded the overall risk, and adding operator level gave a five-factor count with modestly higher discrimination. Together, these results are consistent with the hypothesis that, in this setting, the difficulty of modern video laryngoscopy lay more often after the larynx had been seen than in seeing it—at the stage of tube delivery—although, because this inference conditions on the glottic view, it could not be established directly.

Two of our findings may at first seem to conflict. A poor glottic view carried a higher rate of difficulty, yet most difficult intubations occurred when the view was good. Both observations are valid, and the reconciliation lies in the base rates. The risk of difficulty was several times higher when the view was poor than when it was good (29.3% versus 4.3%), so the view still matters. However, a good view was obtained in almost all patients (92.4%), so, in absolute numbers, good-view cases account for most difficult intubations. The practical implication is that a good view—the reassurance that a video laryngoscope is designed to provide—does not exclude a difficult intubation; visualization and the subsequent delivery of the tube are distinct steps [10]. A recent analysis of Macintosh-type video laryngoscopy likewise found that the laryngeal view alone does not fully explain intubation difficulty, even at favorable Cormack–Lehane grades [29].

That a good view does not guarantee easy tube passage has been reported before, in simulated and critical-care settings [16,17], and one study using an angulated device named this “can see but cannot intubate” gap as an open question [9]. The phenomenon is therefore not itself new; our contribution is to quantify it, and to link it to simple preoperative predictors, in a large otorhinolaryngological cohort with an attempts-based outcome. Consistent with this, recent large real-world series of Macintosh-type video laryngoscopy have separately reported the glottic view, first-attempt success, and the need for adjunct devices, reaffirming that visualization and tube delivery are distinct dimensions of intubation difficulty [30]. Other studies found the opposite pattern—difficulty that rose as the view worsened [8,14]—but those were done in populations in which obtaining the view is itself often difficult, namely an anticipated difficult-airway cohort [8] and cervical spine surgery with manual in-line stabilization [14], whose attempts-based outcome moreover captured tube delivery as well as the view; in such settings a hyperangulated blade, by improving the view, can raise first-attempt success [31]. In our cohort, which was not selected for anticipated difficult airway, a good view was usual, so the difficulty that remained appeared later.

The devices used here spanned a range of blade curvature, from a standard Macintosh profile (AceScope) through a shallow Macintosh blade with an approximately 45° angled tip (UEScope, 66% of intubations) to a steeply angulated GlideScope (1.8%). The two predominant devices, which together accounted for 98% of intubations, had similar difficult-intubation rates (6.3% for the AceScope and 5.9% for the UEScope), indicating that the pattern was not confined to a single blade design; the GlideScope was used in too few patients (34) to support a stable estimate and, having probably been reserved for anticipated difficulty, cannot be compared without confounding, so we do not interpret its rate. Because device choice was not randomized, these device-stratified rates are descriptive only and cannot separate blade geometry from case mix. Whether a hyperangulated blade would reduce, or merely relocate, the difficulty that follows an adequate view is an important question that this observational cohort cannot resolve and that warrants prospective, device-randomized study [32].

An important qualification concerns the operator. A resident operator was one of the independent predictors and one of the count items, and it reflects the process of care rather than a patient characteristic. Within the good-view subgroup, difficulty was more than twice as frequent when a resident performed the intubation (7.6% versus 3.1%), and residents accounted for nearly half (47%) of the good-view difficulties. Consistent with this, the anatomical four-factor count—which contains no operator term—did not, once operator level was included in the same model, remain a predictor within the good-view subgroup (adjusted odds ratio 1.13, 95% CI 0.81–1.57), whereas operator level did (adjusted odds ratio 2.48, 95% CI 1.56–3.96; *p* < 0.001). In other words, once the larynx was well seen, the residual difficulty related more to operator inexperience than to fixed anatomy: a trainee may see the glottis on the monitor yet struggle to advance the tube, lose the view, or be relieved by a supervisor, each of which generates a further attempt. This does not exclude a patient contribution—across the whole cohort the anatomical count did grade risk—but it identifies operator skill as a major, and potentially modifiable, component of the “good view but difficult” pattern. Because this good-view comparison conditions on the glottic view—the same mediator that led us to treat the tube-delivery locus as a hypothesis—the operator association is likewise descriptive rather than causal and may reflect unmeasured common causes of the view and the outcome, such as unrecorded anatomy or how cases are allocated between operators; it too should be read as hypothesis-generating. This direction—more difficulty with less experience—differs from a report in which supervised trainees encountered less difficult video laryngoscopy than independent anesthetists [8]; the sign of an operator effect probably depends on the degree of supervision and on how cases are allocated between grades, which our data could not fully characterize, so the staffing implication is tentative. No single bedside test was strongly predictive; the discrimination of the count was modest, and its Brier score (0.058) barely improved on the no-information value (scaled Brier 0.009), so at the individual level the count adds little. Consistent with this, it did not discriminate better than an established score: the modified El-Ganzouri Risk Index reached a similar apparent area under the curve (0.610 versus 0.596), so the count’s appeal lies in its simplicity and in the nature-of-difficulty observation rather than in superior discrimination. Its high negative predictive value reflects the low event rate rather than strong discrimination, so its potential use is not to rule difficulty in or out but to flag, before induction, patients in whom more than a good view may be required.

These observations generate practical suggestions rather than confirm them. Because a good view did not reliably exclude a difficult intubation, and because in such cases the residual difficulty related more to operator experience than to anatomy, the most consistent suggestion is to have an experienced operator perform, or be immediately available for, the first attempt in higher-risk patients, and to agree on a rescue plan before induction. Preparing for difficult tube delivery—having a stylet or bougie to hand—remains a reasonable but hypothesis-level precaution; a randomized trial found similar first-attempt success with a bougie and a stylet (80.4% versus 83.0%; difference −2.6 percentage points, 95% CI −7.3 to 2.2) [26]. None of these measures was evaluated in this cohort, and each warrants prospective study. The graded association also held in patients with head and neck cancer, but that subgroup was small and the analysis exploratory.

This study has several limitations. First, it was single-center and retrospective, in an East Asian population with a relatively low body mass index and intubated with a mix of non-channeled devices whose blade curvature ranged from a standard Macintosh profile to an approximately 45° angled tip. The specific difficulty rates and the performance of the count may not extend to channeled or steeply hyperangulated practice, or beyond this otorhinolaryngological, East Asian, low-body-mass population, although the qualitative pattern—difficulty persisting despite an adequate view—has been reported across diverse settings and is likely to be more general. Device choice was not randomized, so device geometry and case mix are confounded and cannot be separated. Second, the outcome was defined by the number of laryngoscopic attempts, counted as blade insertions; because a single blade insertion may accompany more than one unsuccessful tube pass, this count is a lower bound on the difficulty of tube delivery, and the true frequency of tube-delivery difficulty is likely higher than reported. Third, the Cormack–Lehane grade was recorded as whole grades (I–IV) without the IIa/IIb subdivision and was developed for direct laryngoscopy, so it may overstate the view obtained at video laryngoscopy and cannot capture finer gradations within a good view. Fourth, the locus of difficulty was inferred, not measured: no variable recorded the reason for a repeated attempt, the depth of neuromuscular blockade, or the stylet or bougie used—the principal determinants of tube delivery—so the attribution to tube delivery rests on the combination of an adequate view and repeated attempts rather than on direct observation. Fifth, operator experience was available only as a binary level (resident versus consultant or fellow); individual operators could not be identified, so we could not account for the clustering of outcomes within operators, and the operator effect should be read as a group-level association whose precision may be optimistic; because the primary four-item count excludes operator level, however, the principal descriptive findings do not depend on this variable.

Further limitations concern the count and the secondary analyses. The items and cut-offs were applied within this dataset and the reported discrimination is apparent (optimism-uncorrected) and modest; the positive predictive value was low and the decision-curve analysis showed net benefit only at very low thresholds; the cohort size was fixed by the availability window of the assessment template rather than sized to current prediction-model criteria; and the subgroup, sensitivity, and comparative analyses were exploratory and hypothesis-generating, not registered in advance, and not corrected for multiplicity. Finally, the 62 patients (2.8%) excluded for incomplete records could not be compared with the analyzed cohort because their partial records were not retained; because these exclusions reflected record completeness rather than patient or outcome characteristics and affected only 2.8% of those screened, the complete-case analysis is unlikely to be materially biased. The count is therefore exploratory and not ready to guide care; prospective, ideally multicenter external validation—recording the stage and reason of difficulty, as planned in emerging trial designs [32]—is required.

## 5. Conclusions

In adults undergoing otorhinolaryngological surgery with non-channeled video laryngoscopy, difficult intubation was uncommon and, when it occurred, arose despite an adequate glottic view in most cases (64% at Cormack–Lehane grade I–II, and 45% at grade I alone). This pattern is consistent with the hypothesis that difficulty lay at the stage of tube delivery rather than visualization, although, because the analysis conditions on the glottic view, the locus of difficulty was inferred rather than demonstrated. A simple count of four anatomical preoperative factors graded the overall risk; once the larynx was well seen, the residual difficulty appeared to relate more to operator experience than to anatomy. These findings suggest that airway preparation may need to anticipate difficult tube delivery, not only a poor view. Because the count was derived and tested in a single cohort, prospective external validation is required before clinical use.

## Figures and Tables

**Table 1:** Patient characteristics by intubation difficulty (*N* = 1932).

Characteristic	All (*N* = 1932)	Easy (*n* = 1812)	Difficult (*n* = 120)	*p*
Age, years	51.0 (16.0)	51.1 (16.0)	49.8 (16.3)	0.419
Male sex	1072 (55.5)	1002 (55.3)	70 (58.3)	0.580
Body mass index, kg/m^2^	24.1 (3.4)	24.1 (3.4)	24.1 (3.7)	0.992
Comorbidity/history				
Cervical spine disease	93 (4.8)	90 (5.0)	3 (2.5)	0.275
Obstructive sleep apnea	59 (3.1)	53 (2.9)	6 (5.0)	0.315
Head and neck cancer	186 (9.6)	171 (9.4)	15 (12.5)	0.346
Neck scar or radiotherapy	7 (0.4)	5 (0.3)	2 (1.7)	0.065
Bedside airway examination				
Modified Mallampati class III–IV	405 (21.0)	371 (20.5)	34 (28.3)	0.053
Inter-incisor distance, cm	4.2 (0.7)	4.2 (0.7)	4.0 (0.6)	<0.001
Thyromental distance, cm	7.6 (1.2)	7.6 (1.2)	7.3 (1.3)	0.005
Limited cervical spine mobility	70 (3.6)	64 (3.5)	6 (5.0)	0.561
Edentulous or denture-wearing	75 (3.9)	74 (4.1)	1 (0.8)	0.085
Loose tooth	229 (11.9)	212 (11.7)	17 (14.2)	0.507
Limited mandibular protrusion	261 (13.5)	237 (13.1)	24 (20.0)	0.044
Snoring	1074 (55.6)	1006 (55.5)	68 (56.7)	0.881
Procedural				
Resident operator	495 (25.6)	452 (24.9)	43 (35.8)	0.011
AceScope	620 (32.1)	581 (32.1)	39 (32.5)	1.000
UEScope	1278 (66.1)	1202 (66.3)	76 (63.3)	0.566
GlideScope	34 (1.8)	29 (1.6)	5 (4.2)	0.087
Laryngoscopy and intubation				
Cormack–Lehane grade III–IV	147 (7.6)	104 (5.7)	43 (35.8)	<0.001
Intubation time, s	41 (34–54)	40 (33–51)	90 (64–137)	<0.001

Values are mean (SD), median (IQR), or *n* (%). Difficult intubation was defined as ≥2 attempts. Continuous variables were compared by Welch’s *t*-test (Mann–Whitney U test for intubation time) and categorical variables by the χ^2^ or Fisher’s exact test; these *p* values are unadjusted and descriptive.

**Table 2:** Univariable and multivariable logistic regression for difficult intubation.

Predictor	Univariable OR (95% CI)	*p*	Adjusted OR (95% CI)	*p*
Age (per year)	1.00 (0.98–1.01)	0.409	0.99 (0.98–1.00)	0.055
Male sex	1.13 (0.78–1.65)	0.517	1.33 (0.89–1.99)	0.159
Body mass index (per kg/m^2^)	1.00 (0.95–1.05)	0.992	1.01 (0.95–1.07)	0.735
Cervical spine disease	0.49 (0.15–1.57)	0.231	—	—
Obstructive sleep apnea	1.75 (0.74–4.15)	0.206	1.71 (0.69–4.24)	0.246
Head and neck cancer	1.37 (0.78–2.41)	0.272	1.50 (0.82–2.73)	0.188
Neck scar or radiotherapy	6.13 (1.18–31.91)	0.031	—	—
Modified Mallampati class III–IV	1.54 (1.02–2.32)	0.042	1.28 (0.83–1.97)	0.257
Inter-incisor distance (per cm)	0.57 (0.43–0.77)	<0.001	0.61 (0.45–0.84)	0.002
Thyromental distance (per cm)	0.79 (0.68–0.93)	0.003	0.82 (0.69–0.97)	0.019
Limited cervical spine mobility	1.44 (0.61–3.39)	0.407	0.94 (0.38–2.35)	0.900
Edentulous or denture-wearing	0.20 (0.03–1.43)	0.109	—	—
Loose tooth	1.25 (0.73–2.12)	0.419	1.30 (0.74–2.28)	0.366
Limited mandibular protrusion	1.66 (1.04–2.65)	0.033	1.37 (0.84–2.25)	0.205
Snoring	1.05 (0.72–1.52)	0.806	1.07 (0.72–1.58)	0.749
Resident operator	1.68 (1.14–2.48)	0.009	1.82 (1.21–2.73)	0.004

OR, odds ratio; CI, confidence interval. The multivariable model included all candidate predictors with at least five events in the exposed group, entered simultaneously (13 predictors; approximately nine events per variable). Cervical spine disease, neck scar or radiotherapy, and edentulous or denture-wearing each had fewer than five events and were assessed univariably only. Univariable odds ratios are from logistic regression and may differ from the descriptive between-group comparisons in Table 1. “—”, not entered in the multivariable model.

## Data Availability

The de-identified individual-level data and the analysis code that support the findings of this study are available from the corresponding author upon reasonable request, subject to the data-governance requirements of the Institutional Review Board of Severance Hospital.

## Supplementary Materials

The following supporting information can be downloaded at https://www.mdpi.com/article/10.3390/jcm15176814/s1, Table S1: Difficult intubation rate by cumulative risk-factor count; Table S2: The coefficients of the underlying four-factor logistic model; Table S3: The sensitivity of the cumulative-count gradient to alternative cut-offs; Table S4: Within-cohort comparison of difficult intubation prediction scores; Figure S1: Calibration of the four-factor anatomical count. Observed difficult intubation rate (with Wilson 95% confidence intervals) plotted against the model-predicted rate, grouped by the number of anatomical risk factors (0, 1, 2, ≥3). Calibration is shown on the development data and is therefore near-ideal by construction and not a validated estimate; the Brier score (0.058) barely improves on the no-information value at this event rate (scaled Brier 0.009); Figure S2: Receiver operating characteristic (ROC) curves for difficult intubation prediction scores within this cohort. The four-factor anatomical count is compared with the modified MACOCHA score and the modified El-Ganzouri Risk Index. Areas under the curve are shown in the legend. The comparators were applied with non-contributing or omitted items as detailed in Table S4; this within-cohort comparison should be interpreted accordingly.

## Abbreviations

The following abbreviations are used in this manuscript:

aOR	Adjusted odds ratio
AUC	Area under the receiver operating characteristic curve
CI	Confidence interval
IQR	Interquartile range
IRB	Institutional Review Board
OR	Odds ratio
SD	Standard deviation
STROBE	Strengthening the Reporting of Observational Studies in Epidemiology
TRIPOD	Transparent Reporting of a multivariable prediction model for Individual Prognosis Or Diagnosis
