# Supplementary material for: Nature and Preoperative Prediction of Difficult Non-Channeled Video-Laryngoscopic Tracheal Intubation in Otorhinolaryngological Surgery: A Retrospective Cohort Study of 1932 Adults
Source: J Clin Med. 2026 Sep 2;15(17):6814. doi: 10.3390/jcm15176814 (PMC13566525; doi:10.3390/jcm15176814)
Supplement: Supplementary file 1 [file jcm-15-06814-s001.zip › jcm-4483798-supplementary.pdf]

Supplementary Materials

Nature and preoperative prediction of difficult non-channeled video-laryngoscopic tracheal intubation in otorhinolaryngological surgery: a retrospective cohort study of 1932 adults

Table S1. Difficult intubation rate by cumulative risk-factor count

Risk factors, n	Patients, n	Difficult intubation, n	Rate, % (95% CI)
0	1184	54	4.6 (3.5–5.9)
1	608	48	7.9 (6.0–10.3)
2	116	15	12.9 (8.0–20.2)
3	21	3	14.3 (5.0–34.6)
4	3	0	0.0 (0.0–56.2)

Trend odds ratio per additional factor 1.63 (95% CI 1.29–2.05; $p < 0.001$) across the full distribution. Four binary anatomical risk factors: inter-incisor distance ≤ 3.0 cm; thyromental distance ≤ 6.0 cm; modified Mallampati class III–IV; limited mandibular protrusion (present in 53, 196, 405, and 261 patients, respectively). The two highest strata are sparse. CI, confidence interval.

Table S2. The Coefficients of the underlying four-factor logistic model

Term	β (log-odds)	Odds ratio (95% CI)	*p*
Intercept	−2.981	—	—
Inter-incisor distance ≤ 3.0 cm	0.819	2.27 (1.02–5.04)	0.044
Thyromental distance ≤ 6.0 cm	0.589	1.80 (1.09–2.99)	0.022
Modified Mallampati class III–IV	0.379	1.46 (0.96–2.22)	0.075
Limited mandibular protrusion	0.384	1.47 (0.91–2.38)	0.117

CI, confidence interval. Predicted probability = $1 / (1 + \exp[-(-2.981 + 0.819 \cdot X_1 + 0.589 \cdot X_2 + 0.379 \cdot X_3 + 0.384 \cdot X_4)])$, where each X_i is 1 if the corresponding binary anatomical finding is present and 0 otherwise. The model was fitted on 120 events (30 events per variable). Because pre-existing rather than data-derived thresholds were used, these estimates carry less in-sample optimism than a fitted-threshold model; external validation is nonetheless required.

Table S3. The Sensitivity of the cumulative-count gradient to alternative cut-offs

Scenario	IID cut-off (cm)	TMD cut-off (cm)	Rates 0/1/2/≥3 (%)	Trend OR (95% CI)	AUC
Classic (primary)	≤ 3.0	≤ 6.0	4.6 / 7.9 / 12.9 / 12.5	1.63 (1.29–2.05)	0.596
Alternative A	≤ 3.5	≤ 6.0	4.5 / 7.5 / 9.1 / 16.3	1.50 (1.23–1.83)	0.595
Alternative B	≤ 4.0	≤ 6.5	4.2 / 5.1 / 9.7 / 13.0	1.50 (1.25–1.80)	0.612
Youden-derived	≤ 4.0	≤ 6.6	4.0 / 5.2 / 9.8 / 12.5	1.51 (1.26–1.80)	0.616

IID, inter-incisor distance; TMD, thyromental distance; OR, odds ratio; CI, confidence interval; AUC, area under the receiver operating characteristic curve. The trend odds ratio per additional factor (approximately 1.5–1.6) and the overall gradient were preserved across cut-off scenarios; the pre-existing 3.0/6.0 cm thresholds were adopted for the primary analysis to avoid the optimism introduced by data-derived cut-offs.

Table S4. Within-cohort comparison of difficult intubation prediction scores

Score	Items applied	AUC (95% CI)
Cumulative risk-factor count	4	0.596 (0.546–0.645)
Modified MACOCHA score	modified Mallampati III–IV, OSA, cervical mobility, mouth opening < 3 cm	0.558 (0.515–0.605)
Modified El-Ganzouri Risk Index	mouth opening, thyromental distance, modified Mallampati, neck movement, prognathism	0.610 (0.558–0.664)

AUC, area under the receiver operating characteristic curve; CI, confidence interval; OSA, obstructive sleep apnea. In this within-cohort comparison the four-factor count had discrimination similar to the modified MACOCHA and modified El-Ganzouri scores, and all three were modest; the comparator areas are shown descriptively, without paired significance testing, because the count's apparent area was derived in this cohort and is not compared on equal terms. The comparators are imperfect: the modified MACOCHA score omits the coma and severe-hypoxemia items (inapplicable to elective surgery), and its non-anesthesiologist-operator item contributed 0 to every patient because all intubations were performed by anesthesiologists; the modified El-Ganzouri Risk Index omits the prior-difficult-intubation and body-weight items, neither retrievable from this dataset.

Supplementary Figure Legends

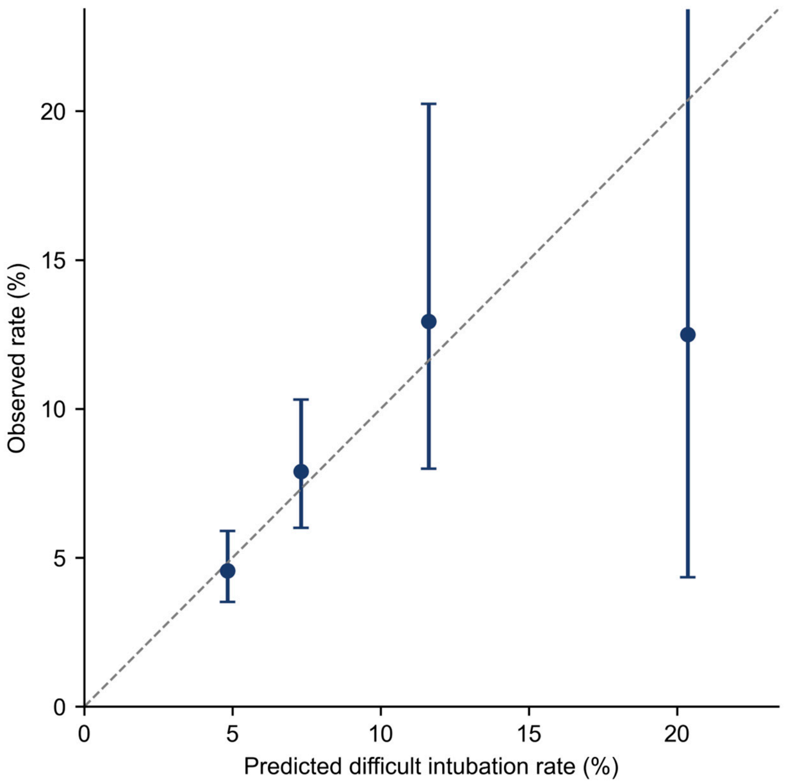

Figure S1. Calibration of the four-factor anatomical count. Observed difficult intubation rate (with Wilson 95% confidence intervals) plotted against the model-predicted rate, grouped by the number of anatomical risk factors (0, 1, 2, ≥ 3). Calibration is shown on the development data and is therefore near-ideal by construction and not a validated estimate; the Brier score (0.058) barely improves on the no-information value at this event rate (scaled Brier 0.009).

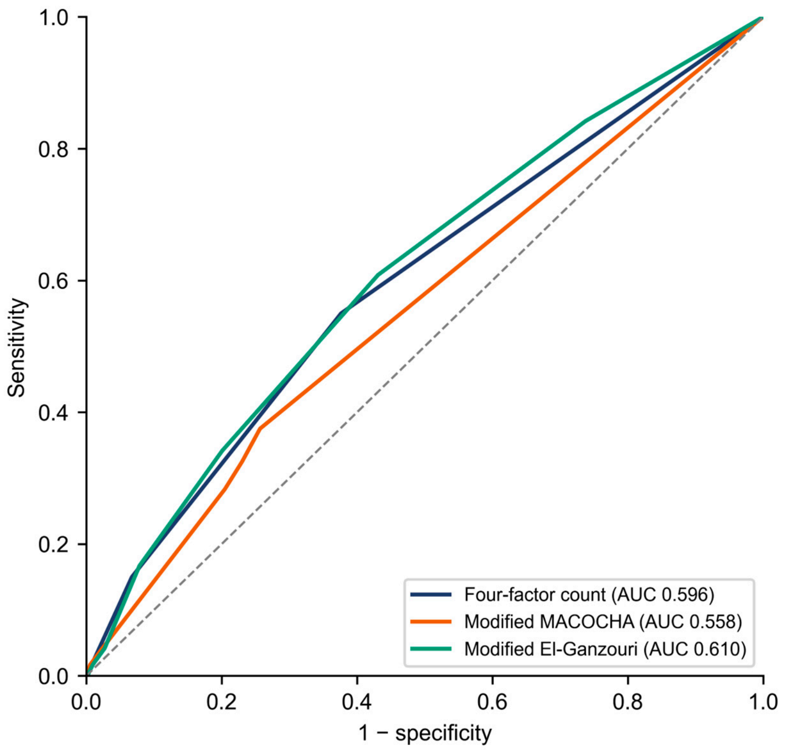

Figure S2. Receiver operating characteristic (ROC) curves for difficult intubation prediction scores within this cohort. The four-factor anatomical count is compared with the modified MACOCHA score and the modified El-Ganzouri Risk Index. Areas under the curve are shown in the legend. The comparators were applied with non-contributing or omitted items as detailed in Table S4; this within-cohort comparison should be interpreted accordingly.
